# Research Progress of Osteoarthritis Treatment by Low Intensity Pulsed Ultrasound

**DOI:** 10.1002/smmd.70003

**Published:** 2025-05-16

**Authors:** Mengtong Guan, Xinyu Zhang, Xinhe Li, Bo Liao, Wang Han, Jindong Tan, Zijie Wang, Lichen Wang, Jieliang Shen, Xiaoyu Han, Dingqun Bai

**Affiliations:** ^1^ Department of Rehabilitation Medicine Key Laboratory of Physical Medicine and Precision Rehabilitation of Chongqing Municipal Health Commission The First Affiliated Hospital of Chongqing Medical University Chongqing China; ^2^ State Key Laboratory of Ultrasound in Medicine and Engineering Chongqing Medical University Chongqing China; ^3^ Stevens Institute of Technology Hoboken New Jersey USA; ^4^ Department of Rehabilitation Medicine Bishan Hospital of Chongqing Medical University Chongqing China

**Keywords:** combination therapy, low intensity pulsed ultrasound, osteoarthritis, treatment mechanism

## Abstract

With the intensification of global population aging, osteoarthritis (OA) has emerged as a major socioeconomic burden requiring urgent therapeutic interventions. Low‐intensity pulsed ultrasound (LIPUS), a non‐invasive physical therapy modality, delivers pulsed acoustic energy to target tissues with negligible thermal effects. Accumulating evidence from preclinical studies and randomized controlled trials has demonstrated its potential to decelerate OA progression. This systematic review synthesizes current knowledge on LIPUS‐mediated OA management, elucidates mechanistic pathways through biomechanical and molecular analyses, strategies combining LIPUS with biomaterials to improve its efficacy, evaluates clinical translation challenges, and proposes standardized treatment protocols to optimize therapeutic outcomes.

## Graphical abstract

As a non‐invasive therapeutic modality, LIPUS demonstrates potential in mitigating OA progression. This review synthesizes current evidence of OA pathophysiology, summarizes the mechanism of LIPUS treatment of OA, and proposes synergistic strategies to advanced OA treatment.

**Summary**

- It illustrates the pathological change of each structure during OA progression.
- IIt reviews the therapeutic mechanism of LIPUS for treating OA based on structures of the joint.
- IIt summarizes and prospects LIPUS combination with biomaterials to further enhance the therapeutic effect.

## 1 Introduction

Osteoarthritis (OA) is a degenerative disease involving the entire joint, which is the leading cause of disability in the elderly and severely influences the quality of life of patients. With the intensification of aging population, it brings a huge burden to the socio‐economic system [1]. OA‐induced knee pain significantly restricts ambulation, contributes to the development of depressive symptomatology, and substantially compromises overall quality of life [2]. Therefore, the search for effective OA treatment methods is imminent. The pathological characteristics are mainly manifested in the degeneration of articular cartilage, synovial inflammation, subchondral bone remodeling, meniscal damage, and osteophyte formation [3, 4], even leading to joint dysfunction, pain, stiffness, limited function, and affecting the ability of daily activities to a certain extent [5]. Due to the complexity of its etiology and pathology, there is currently no drug available with a significant therapeutic effect for OA. The role of drug treatment is only limited to controlling pain and inflammation. What's more, late‐stage patients can only be intervened through invasive methods such as intra‐articular injection or even joint replacement [6]. Projections indicate a 276% and 208% increase in knee and hip OA‐related joint replacements among patients under 55 years old by 2030 [7]. Given the finite longevity of prosthetic implants coupled with the higher functional demands of younger OA patients, this demographic faces a 35% lifetime risk of revision surgery—a fivefold increase compared with individuals over 75 years [8]. The resultant economic burden encompasses not only substantial direct medical expenditures but also significant indirect costs associated with productivity losses and long‐term disability management. Therefore, the development of a non‐invasive, safe, and effective therapeutic strategy for OA has become an urgent clinical imperative.

Ultrasound, as a mechanical wave, applies controlled mechanical forces to cellular structures while enabling energy transfer to the surrounding microenvironment. In tissue engineering, acoustic radiation forces enable the mobilization, alignment, and aggregation of cellular or particulate components. Furthermore, viscous dissipation of acoustic energy induces acoustic streaming, thereby generating directional fluid motion within liquid media. Such hydrodynamic effects enhance nutrient and cellular transport via drag forces, while simultaneously redistributing or aggregating cells in regions of divergent flow velocities. Collectively, these mechano‐acoustic phenomena drive the fabrication of functionally organized living tissues [9]. Ultrasound therapy has a long‐standing history in medicine. Unlike high‐intensity ultrasound that generates thermal effects, Low‐intensity pulsed ultrasound (LIPUS) has garnered increasing attention as a non‐invasive and safe physical therapy modality; it generates minimal thermal effects while maintaining acoustic energy transmission to target tissues in a pulsed wave pattern, providing non‐invasive physical stimulation to therapeutic tissues [10]. It primarily influences cellular activities through its mechanical effects, such as altering the flow of fluid around cells, promoting the absorption of nutrients and the expulsion of metabolic waste, thereby enhancing the regenerative capacity of cellular tissues [11, 12]. It plays anti‐inflammatory and reparative roles in the treatment of soft tissue injuries, fractures, and non‐union, achieving therapeutic goals [10, 13, 14]. Recently, an increasing number of basic research and clinical studies have shown that LIPUS can alleviate the progression of OA, offering a novel possibility for the non‐invasive treatment of osteoarthritis.

**FIGURE 1:**
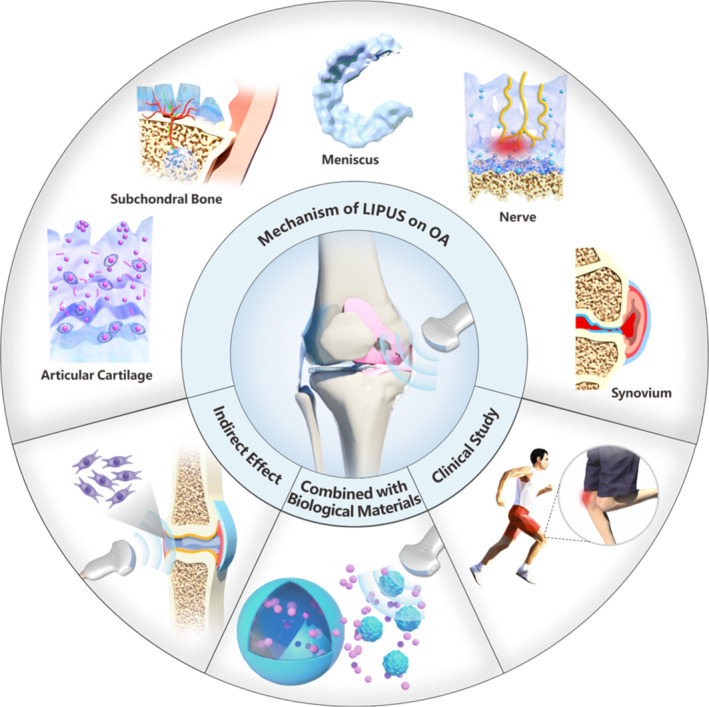
Schematic illustration of LIPUS treating OA.

This systematic review integrates multi‐decadal experimental and clinical evidence on the therapeutic implementation of LIPUS in OA management. Our analysis meticulously examines LIPUS applications, therapeutic efficacy, and mechanistic interactions with OA‐affected joint structures, encompassing articular cartilage, synovium, subchondral bone, and menisci. By systematically elucidating LIPUS‐mediated biological pathways, this work ultimately aims to refine treatment protocols and accelerate clinical translation through mechanistic optimization (Figure 1).

## 2 Low‐Intensity Pulsed Ultrasound (LIPUS)

Ultrasound (US), defined as mechanical oscillations with frequencies beyond human auditory perception (> 20 kHz), has gained widespread biomedical and industrial applications attributed to its precise spatial control, non‐destructive nature, and superior soft tissue penetrability [15, 16]. Propagation of ultrasound requires an elastic medium, achieved through transducer‐mediated energy conversion that transforms electrical impulses into mechanical vibrations. These vibrations generate alternating compression‐rarefaction wavefronts that transmit energy through the medium via particle displacement [17]. Ultrasound can be categorized based on intensity into high‐intensity and low‐intensity ultrasounds. At high intensities, thermal and cavitation effects predominate, potentially leading to instantaneous cell death. Cavitation is produced in gas nuclei stabilized within the tissue that expand to form a bubble when the frequency‐dependent negative pressure and the mechanical index exceed a certain threshold, which is known to cause irreversible damage by mechanically disrupting cell membrane permeability and altering the structure of cells [18]. Specifically, the strength of propagating shock waves in multi‐bubble systems resulting from cavitation implosions is dependent primarily on the speed of sound in the liquid and interfacial properties such as surface tension, viscosity, gas content, vapor pressure and density [19]. The most prevalent application of high‐intensity ultrasound is treating malignancy by using High‐Intensity Focused Ultrasound, as knows HIFU [18, 20]. Conversely, low‐intensity ultrasound is generally considered beneficial and plays a role in tissue repair, physical therapy, sonophoresis, and gene therapy, among other applications [21].

**FIGURE 2:**
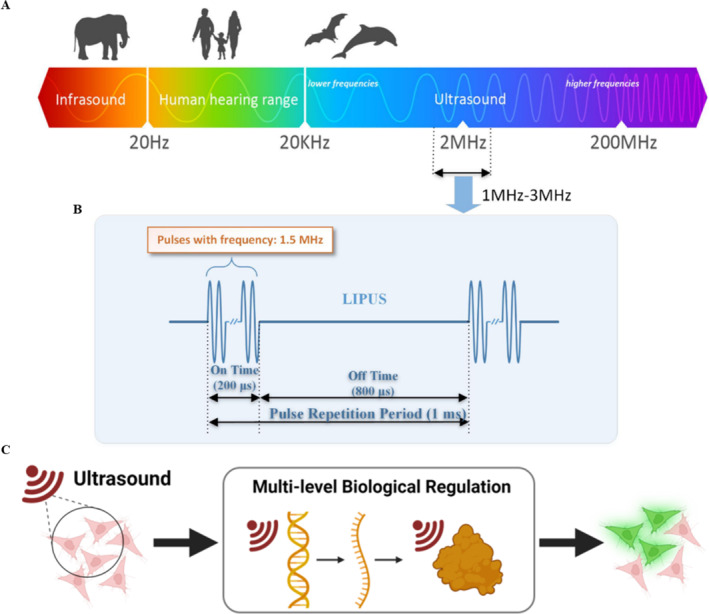
Schematic diagram of LIPUS parameters and mechanisms. (A) The frequency range of ultrasound [10]; (B) Schematic diagram of LIPUS parameters [10]; (C) Schematic diagram of LIPUS mechanisms [15]. Reproduced with permission [10]. Copyright 2019, IEEE. Reproduced under terms of the CC‐BY license [15]. Copyright 2024, The Authors, published by Wiley‐VCH GmbH.

In recent years, the physical therapeutic agent widely applied in tissue repair is LIPUS, which typically employs frequencies ranging from 1 to 3 MHz. The spatial average temporal average (SATA) intensity of ultrasound is in the range of 0.02–1 W/cm^2^(Figure 2A, B), and the duration of treatment is 5–20 min per day [10]. The therapeutic effect is primarily exerted through mechanical effects [22]. US emits periodic mechanical sound waves inducing vibrations and collisions that penetrate targeted tissues, modulates cellular metabolism, accelerates tissue healing processes, ameliorates cellular ischemia and hypoxia, ultimately enhancing tissue nutrition and promoting tissue repair (Figure 2C) [15]. It is worth noting that LIPUS elicits minimal thermal effects or even non‐thermal [23]. However, the mechanistic transduction of mechanical stimuli into biochemical signaling is not well understood.

## 3 Pathological Mechanism of Osteoarthritis

**FIGURE 3:**
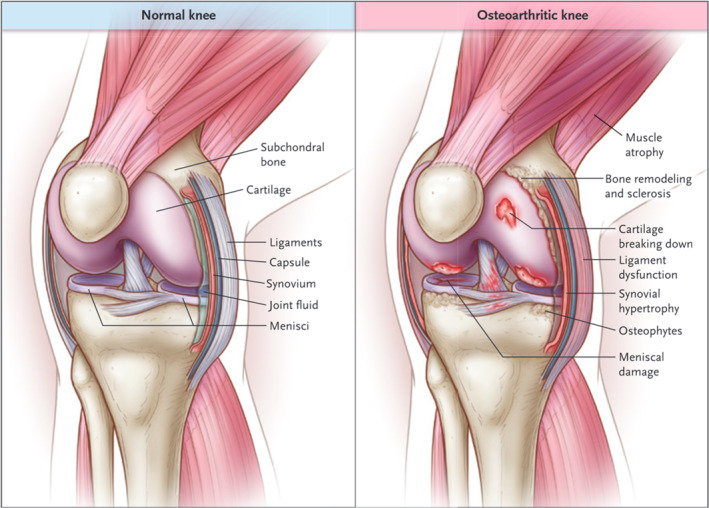
Comparison of normal and osteoarthritic knees. Reproduced with permission [3]. Copyright 2021, Massachusetts Medical Society.

Osteoarthritis (OA) is a form of joint damage caused by abnormal stress on the joint or its surrounding tissues [3], with knee osteoarthritis being the most prevalent [24]. The clinical manifestations of OA primarily include pain, joint stiffness, muscle weakness, and restricted joint mobility [3], which not only severely affects the quality of life of OA patients but also arouses an enormous burden on society. Factors that have been correlated with an elevated risk of knee OA include advanced age, female gender, overweight or obesity, knee injury, occupational factors, and varus or valgus knee alignment. In addition, recreational physical activity does not increase the risk [25, 26]. Pathological changes observed in OA joints encompass progressive loss and destruction of cartilage, remodeling of subchondral bone, exception formation of osteophytes, inflammation of the synovium, hypertrophy of the joint capsule, degeneration of ligaments, surrounding muscle, and meniscus, which affect all joint structures [4, 27]. Although cartilage destruction is a central feature of these pathological changes, OA affects the entire joint (Figure 3) [3]. However, the molecular mechanisms underlying the onset and progression of OA require more in‐depth investigation.

## 4 The Mechanism of LIPUS Treating OA Directly

In recent years, LIPUS has been comprehensively applied in clinic for a variety of conditions, such as treating soft tissue pain (chronic prostatitis and chronic pelvic pain syndrome) [28], promoting the healing of fresh fractures and non‐union [29, 30], alleviating pain caused by orthodontic separation [31], reducing pulmonary and serum inflammatory factor levels of COVID‐19 patients in hospital [32], and providing therapeutic effects for vitiligo on the trunk [33]. Preliminary clinical trials have also confirmed that LIPUS can slow the progression of knee osteoarthritis (OA), alleviate patient pain, and to some extent improve the quality of life of patients, although its mechanism of action is not yet fully understood [34, 35, 36]. However, reports on the therapeutic application of LIPUS for OA remain primarily limited to preclinical studies. A growing body of basic research has demonstrated that LIPUS significantly alleviates OA symptoms, partially elucidating its mechanisms of action. This review synthesizes current basic research on OA‐related pathological alterations in articular cartilage, synovium, subchondral bone, and meniscus, while examining the associated therapeutic mechanisms of LIPUS. Additionally, we present the latest evidence‐based advances in this field and outline potential future research directions.

### 4.1 LIPUS Promotes Cartilage Matrix Synthesis

**FIGURE 4:**
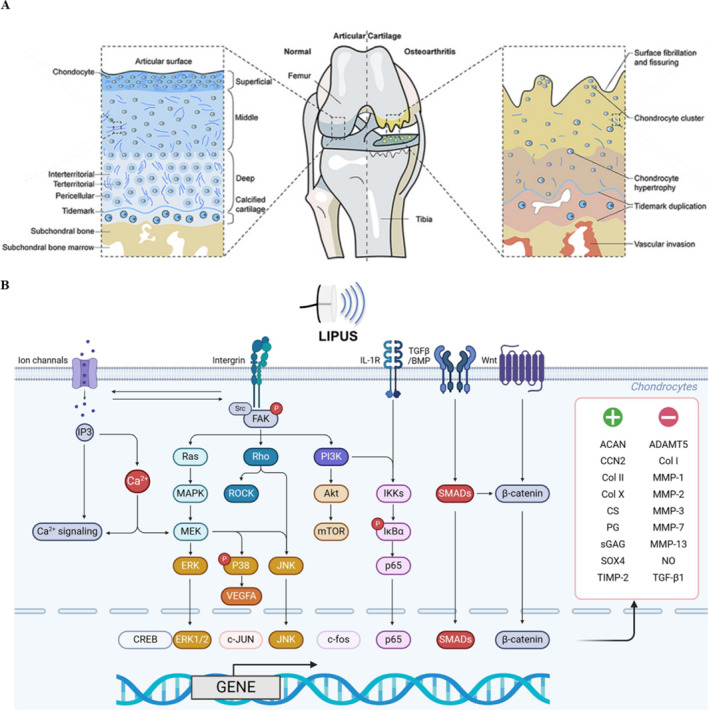
LIPUS promotes cartilage matrix synthesis. (A) Comparison of normal and pathological joint surface structures [38]; (B) LIPUS regulates chondrocyte function via multiple pathways [22]. Reproduced under terms of the CC‐BY license [22]. Copyright 2024, The Authors, published by Frontiers Media S.A. Reproduced with permission [38]. Copyright 2021, Elsevier Ltd.

Articular cartilage is a type of specialized connective tissue in diarthrodial joints, constituting one of the most important structural components of the joint [37]. The articular surface consists of four main elements: articular cartilage (containing chondrocytes), the tidemark (the boundary between calcified and uncalcified cartilage), calcified cartilage, and subchondral bone. As arthritis progresses, the articular surface exhibits fragmentation of articular cartilage, proliferation and enlargement of chondrocytes, repeated advancement of the tidemark, expansion of the calcified cartilage area, and is accompanied by vascular invasion (Figure 4A) [38]. At the microscopic level, articular cartilage is further divided into superficial, middle, and deep zones, each exhibiting characteristic extracellular matrix (ECM) compositions reflective of mechanical forces [37]. Chondrocytes and type II collagen fibers in the superficial zone are oriented transversely, dispersing shear forces during joint loading, while the presence of lubricin further alleviates frictional stress. In the middle zone, type II collagen fibers resist compressive and shear forces from multiple directions, as indicated by their random arrangement. The thick collagen fibers in the deep zone are oriented vertically to resist compressive loads, and the high concentration of proteoglycans in this area helps maintain hydration. As arthritis progresses, functional ECM deteriorates, leading to loss of tissue hydration and the production of abnormal fibrous ECM components and fissures, along with pathological changes in chondrocyte phenotype, including aggregation, hypertrophy, senescence, and inflammation [39].

Articular cartilage is a structure composed of the only cell type chondrocytes and the extracellular matrix (ECM) secreted by itself, covering the surfaces of synovial joints to absorb and cushion stress as well as to reduce friction. Due to its avascular nature, its capacity for self‐repair is extremely limited [37]. Chondrocytes are the sole cellular components encapsulated within the ECM secreted by themselves [40]. Mechanical stimuli transduced by chondrocytes can lead to a series of intracellular biological responses. Moderate mechanical stimulation helps maintain the homeostasis of cartilage, while excessive stimulation can lead to cartilage damage, which is closely related to the occurrence and progression of OA. The integrity of articular cartilage depends on the normal function of chondrocytes and appropriate biomechanical stimulation [41, 42, 43]. Chondrocytes contribute to the formation of the extracellular matrix by synthesizing collagen (primarily Collagen II) and proteoglycans, and they also secrete enzymes that degrade the matrix, such as matrix metalloproteinases (MMPs) family members, such as MMP‐1, MMP‐3, MMP‐9, and MMP‐13, with MMP‐13 having the strongest degradative effect, collectively maintaining a dynamic balance between ECM synthesis and degradation. However, during OA, the level of matrix degradation significantly exceeds that of synthesis, leading to the loss of articular cartilage. Concurrently, OA chondrocytes exhibit abnormal hypertrophic differentiation, ultimately leading to chondrocyte apoptosis [4, 39]. Therefore, maintaining the regular function and the structural integrity of articular cartilage and maintaining the balance between synthesis and degradation of the ECM is a signal for preventing OA. Numerous previous basic research studies on LIPUS therapy for OA have focused on maintaining this balance as their theory foundation.

A previous study has demonstrated that LIPUS regulates chondrocyte function through multiple pathways (Figure 4B) [22]. Current research primarily focuses on cartilage treatment by LIPUS (Table 1). For example, Sang et al. [44] utilized chondrocyte cell lines C28/I2 and CHON‐001 and treated them with LIPUS (parameters: frequency 1.5 MHz, intensity 50 mW/cm^2^ and 100 mW/cm^2^, duty cycle 20%, single session), discovering that LIPUS treatment at both intensities could promote chondrocyte proliferation and differentiation, thereby enhancing the synthesis of the cartilage matrix. This effect was partially mediated through the Focal Adhesion Kinase (FAK) pathway. In the in vivo portion of the study, male C57 mice aged 8 weeks were subjected to an Anterior Cruciate Ligament Transaction (ACLT) model of traumatic osteoarthritis, and postoperatively treated with LIPUS (parameters: frequency 3 MHz, intensity 40 mW/cm^2^, duty cycle 20%, 20 min per session, once per day) for 8 weeks. The results recommended that the load‐bearing capacity of the surgical group was obviously lower than that of the LIPUS‐treated group. Furthermore, the articular cartilage degeneration scores of the harvested specimens were significantly higher in the OA group compared with the LIPUS‐treated ones. In this study, different treatment parameters were used for mice and chondrocytes, but no explanation was provided for this discrepancy. Our research group previously established a traumatic OA model in mice using Destabilization of Medial Meniscus (DMM) and treated it with LIPUS (parameters: frequency of 1.5 MHz, intensity of 30 mW/cm^2^, duty cycle of 20%, 20 min per session, once per day, 6 days per week) for consecutive 2 weeks. After 12 weeks, the joint specimens were scored referring to the Osteoarthritis Research Society International (OARSI) system, showing that LIPUS significantly mitigated the destruction of articular cartilage. This effect was partially dependent on the reduction of Vascular Endothelial Growth Factor A (VEGFA) levels in OA cartilage tissue. In vitro experiments also confirmed that LIPUS could decrease VEGFA levels in OA chondrocytes and promote matrix synthesis [45]. Additionally, a study on rat temporomandibular joint arthritis [46] treated rats under Unilateral Occlusal Trauma (UOT) with LIPUS (parameters: frequency 1 MHz, intensity 100 mW/cm^2^, duty cycle 20%, and the pulse repetition frequency of 100 Hz, 20 min per session, once per day, 5 days a week) for 4 weeks, which could promote matrix synthesis in rat temporomandibular joint cartilage. They also utilized the same LIPUS parameters to treat the inflammatory chondrocytes pretreated by IL‐1β in vitro for 1 week, to further clarify the effect of promoting matrix synthesis was associated with the specific upregulation of ZNT‐9 expression. Sekino et al. [47] conducted in vitro studies using the mouse chondrogenic precursor cell line ATDC5 and found that LIPUS (parameters: frequency 1.5 MHz, duty cycle 20%, intensity 30 or 60 mW/cm^2^, 20 min/d, for 7d) promoted the synthesis of the cartilage matrix by activating the ERK1/2 signaling and downregulating the expression of matrix‐degrading enzyme MMP‐13, further delaying endochondral ossification. Moreover, Uddin, Richbourgh et al. [11] used IL‐1β‐treated human cartilage explants and chondrocytes to create an OA model in vitro, and treated it with LIPUS (parameters: frequency 1.0 MHz, intensity 30 mW/cm^2^, duration 20 min × 7d), indicating that it could alleviate the degradation of proteoglycans in OA chondrocytes. This demonstrated that LIPUS reduced the expression of catabolic genes including MMP13 and ADAMT5 in chondrocytes partially by inhibiting IL‐1β induced phosphorylation of NFkB‐p65 and IkBa. Nishida et al. [48] treated chondrocytes with LIPUS (parameters: frequency 3.0 MHz, intensity 30, 45, 60 mW/cm^2^, duration 20 min, single session), and the results showed that LIPUS promoted chondrocyte differentiation and the expression of extracellular matrix. This effect was mainly due to LIPUS promoting calcium influx and activating the p38‐MAPK and ERK1/2 signaling pathways, a process dependent on CCN2. Differently, LIPUS was also able to upregulate the mRNA levels of matrix degrading enzyme MMP13 simultaneously in this study, which may be related to the use of different parameters or cell models. The latest study used the P2 generation of rat primary chondrocytes induced by inflammation and treated them with LIPUS (parameters: frequency 1.5 MHz, intensity including 30, 45, 60 mW/cm^2^, for 10, 20, 30 min per session, single session, duty cycle 20%). The cells were collected immediately after ultrasound treatment and found that LIPUS alleviated the progression of OA in vitro by restoring the impaired autophagy levels in chondrocytes, with the optimal parameter combination being an intensity of 30 mW/cm^2^ and a treatment duration of 20 min. In vivo experiments involved SD rats that underwent DMM surgery to establish a traumatic OA model, and postoperatively received LIPUS treatment (parameters: frequency 1.5 MHz, intensity 30 mW/cm^2^, duty cycle 20%, 20 min per session, once per day, 5 days a week) for 6 weeks. The experiment further confirmed that LIPUS alleviated the progression of experimental OA in rats through the YAP‐related signaling pathway. The latest study used the P2 generation primary chondrocytes of rats induced by inflammation and treated them with LIPUS (parameters: frequency 1.5 MHz, intensity 30, 45, 60 mW/cm^2^, duty cycle 20%, 10, 20, 30 min per session, single session). The cells were collected immediately after ultrasound treatment and found that LIPUS alleviated the progression of OA in vitro by inhibiting the YAP–RIPK1–NF‐κB axis and restoring the impaired autophagy levels in chondrocytes, with the optimal parameter combination being an intensity of 30 mW/cm^2^ and a treatment duration of 20 min. In vivo experiments involved SD rats that underwent DMM surgery to establish a traumatic OA model, and postoperatively received LIPUS treatment (parameters: frequency 1.5 MHz, intensity 30 mW/cm^2^, duty cycle 20%, 20 min per session, once per day, 5 days per week) for 6 weeks. The results further confirmed that LIPUS alleviated the progression of experimental OA in rats through the YAP‐related signaling pathway [49]. Our recent study further confirmed that LIPUS significantly mitigated the destruction of articular cartilage. Demonstrated that LIPUS activates calcium signaling in chondrocytes and alleviates OA partially by promoting chondrocyte autophagy in a calcium‐dependent manner. Furthermore, the mechanosensitive ion channel TRPV4 mediates the regulatory effects of LIPUS on chondrocytes [50].

**TABLE 1:** The effects of LIPUS on cartilage.

Author(Year)	Model	Parameter	Outcome	Ref.
Sang et al. (2021)	C28/I2 and CHON‐001 cell lines. Male C57BL/6 mice ACLT OA model.	1.5 MHz, 50 mW/cm^2^ or 100 mW/cm^2^ for 10 min. 3 MHz, 40 mW/cm^2^, 20 min/d for 8w.	LIPUS alleviates OA condition and promotes chondrocyte proliferation and differentiation by activating FAK signaling.	[44]
Guan et al. (2020)	Mouse primary chondrocytes pretreated by IL‐1β. Male C57BL/6 mice DMM OA model.	1.5 MHz, 30 mW/cm^2^ for 20 min. 1.5 MHz, 30 mW/cm^2^, 20 min/d for 2w.	LIPUS protects articular cartilage by inhibiting VEGFA expression, which mainly through inhibiting p38 MAPK signaling.	[45]
He et al. (2021)	Rat primary chondrocytes pretreated by IL‐1β. Rat UOT TMJ‐OA model.	1 MHz, 100 mW/cm^2^, 20 min/d for 1w. 1 MHz, 100 mW/cm^2^, 20 min/d for 4w.	LIPUS protects chondrocytes by increasing the expression of aggrecan through ZNT‐9.	[46]
Sekino et al. (2018)	Mouse chondroprogenitor cell line ATDC5, stimulated by ITS for 14 days.	1.5 MHz, 30 or 60 mW/cm^2^, 20 min/d for 7d.	LIPUS induces collagen synthesis and the remodeling of aggrecan via the activation of ERK1/2.	[47]
Uddin et al. (2016)	Human cartilage explants and chondrocytes pretreated by IL‐1β.	1 MHz, 30 mW/cm^2^, 20 min/d for 7d.	LIPUS reduces expression of MMP13 and ADAMT5 in chondrocytes through inhibiting IL‐1β induced phosphorylation of NFkB‐p65 and IkBa.	[11]
Nishida et al. (2016)	Chondrocytic cell line (HCS)‐2/8 and primary rat chondrocytes.	3 MHz, 30, 45 or 60 mW/cm^2^ for 20 min.	LIPUS promotes calcium influx, activates the p38‐MAPK and ERK1/2 signaling, and further facilitates chondrocyte differentiation.	[48]
Pan et al. (2024)	P2 generation of rats primary chondrocytes pretreated by IL‐1β. SD rat DMM OA model.	1.5 MHz, 30, 45 or 60 mW/cm^2^ for 10, 20, 30 min. 1.5 MHz, 30 mW/cm^2^, 20 min/d for 6w.	LIPUS alleviated OA by inhibiting the YAP–RIPK1–NF‐κB axis and restoring the impaired autophagy levels in chondrocytes, with the optimal intensity of 30 mW/cm^2^ for 20 min.	[49]
Guan et al. (2025)	Mouse primary chondrocytes pretreated by IL‐1β. Male C57BL/6 mice DMM OA model.	1.5 MHz, 30 mW/cm^2^ for 20 min. 1.5 MHz, 30 mW/cm^2^, 20 min/d for 2w.	LIPUS activates calcium signaling in chondrocytes and alleviates OA partially by promoting chondrocyte autophagy in a calcium dependent manner. The mechanosensitive ion channel TRPV4 mediates this effect.	[50]

Collectively, modulation of LIPUS treatment parameters demonstrates moderate efficacy in enhancing osteoarthritic cartilage matrix synthesis and attenuating articular cartilage degeneration. Extensive research has characterized intracellular signaling cascades activated in chondrocytes post‐LIPUS stimulation. Despite the established mechanosensitivity of chondrocytes, the precise mechanotransduction pathways through which LIPUS transduces biomechanical signals into intracellular responses remain poorly elucidated. A systematic investigation of these spatiotemporal mechanobiological interactions represents a critical scientific priority.

### 4.2 LIPUS Alleviate Synovial Inflammation

Contemporary understanding of arthritic pathology has progressively expanded beyond a singular focus on articular cartilage, recognizing the synovium as a pivotal contributor to OA progression through bidirectional crosstalk with cartilage. Emerging evidence underscores the pathophysiological significance of low‐grade synovitis and chronic synovial hyperplasia, which are now established as pathognomonic histopathological features of OA. These synovial abnormalities are closely associated with clinical manifestations such as joint pain and exert profound clinical implications for functional joint deterioration [51, 52].

**FIGURE 5:**
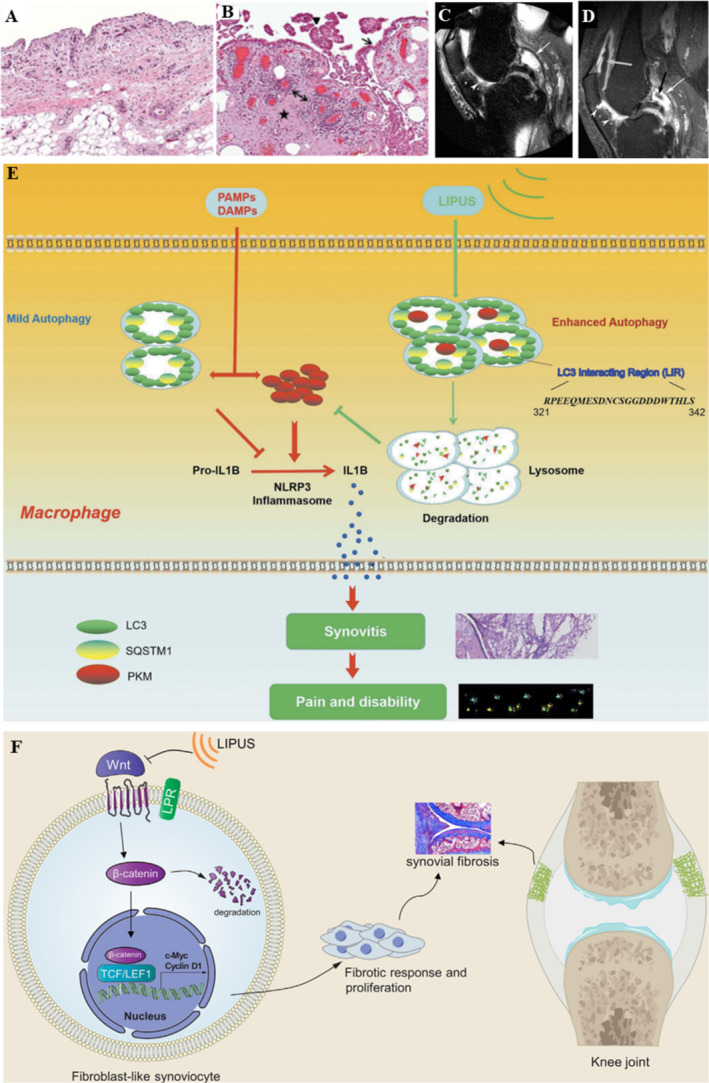
LIPUS relieves synovial inflammation. (A) The H&E staining of Normal synovial [53]; (B) The H&E staining of OA synovial [53]; (C) Imaging manifestations of joint sagittal synovitis (non‐CE‐MRI) [54]; (D) Imaging manifestations of joint sagittal synovitis (CE‐MRI) [54]; (E) LIPUS mainly improves synovial inflammation by enhancing autophagy of macrophages [55]; (F) LIPUS alleviates synovial fibrosis in OA by inhibiting Wnt/β ‐ catenin signaling transduction [56]. Reproduced with permission [53]. Copyright 2012, Elsevier Inc. Reproduced with permission [54]. Copyright 2011, W.B. Saunders Ltd. Reproduced with permission [55]. Copyright 2020, Landes Bioscience. Reproduced with permission [56]. Copyright 2021, Elsevier (Singapore) Pte Ltd.

Structurally, the synovium is a loosely arranged connective tissue that lines the joint cavity. It is composed of loose connective tissue and the lining layer, which primarily contains synovial fibroblasts and resident macrophages (Figure 5A) [53]. They secrete synovial fluid, delivering nutrients and oxygen to other joints. On the other hand, the synovial fluid removes metabolic waste and matrix degradation products [57]. In patients with OA, the synovium may exhibit signs of hypertrophy, inflammatory cell infiltration, hyperplasia, fibrosis, increased thickness, and neovascularization (Figure 5B). Histopathological sections stained with H&E revealed synovial lining cell hyperplasia (arrows), villous hyperplasia (triangles), fibrosis (stars), and perivascular mononuclear cell infiltration (double‐headed arrows) [53]. Magnetic resonance imaging (MRI) offers 3D or tomographic viewing perspectives, providing cross‐sectional images of anatomical structures. Non‐contrast‐enhanced MRI (non‐CE‐MRI) in the sagittal plane of the joint shows high signal fluid equivalent signal changes visible in the posterior cruciate ligament (white arrows), and discrete high signals around the Hoffa's fat pad (white triangles), which are considered signs of grade 2 synovitis (Figure 5C). In addition, in CE‐MRI (Figure 5D) of the sagittal plane of the joint, significant synovitis (white arrows) and a few synovial fluids (black arrows) were observed in the posterior region, and around the Hoffa's fat pad (white triangle) also showed obvious synovitis like features. Only CE‐MRI can distinguish enhanced synovitis from non‐enhanced fluid accumulation above and behind the patella (gray and black arrows) [54].

Within the joint inflammatory niche, synovium and cartilage coexist in a pathologically coupled microenvironment, wherein synovial homeostasis critically regulates cartilage degeneration and repair processes [58]. Cartilage‐derived catabolic products are released into the synovial fluid and phagocytosed by synovial macrophages, thereby amplifying synovitis. Reciprocally, activated synovial inflammatory cells secrete elevated levels of pro‐inflammatory cytokines (e.g., IL‐1, TNF‐α, IL‐6, IL‐8), which drive excessive protease production and accelerate cartilage degradation. This reciprocal crosstalk establishes a self‐perpetuating cycle between synovial inflammation and cartilage destruction [59].

For example, B. Zhang et al. [55] established a traumatic arthritis model using 8‐week‐old male C57 mice with DMM and a synovitis model using an air pouch. Following two consecutive weeks of LIPUS treatment (parameters: frequency 1.5 MHz, intensity 30 mW/cm^2^, duty cycle 20%, 20 min per session, once per day, 6 days per week). They also utilized human THP‐1 monocytes and RAW 264.7 cells pretreated with LPS and ATP, following LIPUS treatment (parameters: frequency 1.5 MHz, intensity 30 mW/cm^2^, duty cycle 20%, 20 min). The experiments both in vivo and in vitro demonstrated that LIPUS promotes autophagic degradation of PKM2 protein in macrophages in an SQSTM1‐dependent manner, inhibiting the maturation of IL‐1β production and thereby alleviating synovial inflammation, leading to improved gait in postoperative mice (Figure 5E). Moreover, Feltham et al. [60] established a post‐traumatic OA model in rats with IAF (Intra‐articular Fracture) and applied LIPUS treatment (parameters: frequency 1.5 MHz, intensity 30 mW/cm^2^, duty cycle 20%, 20 min per session, once per day) for 2 weeks post‐surgery. Though there was no statistically difference in synovial scores between the two groups, the LIPUS treatment group showed significantly less leukocyte infiltration in the synovium, particularly CD3^+^ lymphocytes and CD68^+^ macrophages. Concurrently, LIPUS reduced the expression of the inflammatory cytokine IL‐1β in the joint fluid. Another study by our group [56] in a mouse DMM model of traumatic OA found that 2 weeks of LIPUS treatment (parameters: frequency 1.5 MHz, intensity 30 mW/cm^2^, duty cycle 20%, 20 min per session, once per day, 6 days per week) could alleviate synovial fibrosis in the knee joints of postoperative mice. Furthermore, FLSs were used for mechanism investigation in OA patients; we found an effect associated with the inhibition of synovial fibroblast proliferation and dependent on the Wnt/β‐catenin signaling pathway, which provides a scientific basis for the application of LIPUS in improving joint stiffness and pain in OA patients (Figure 5F). In addition, Itaya et al. [13] established a joint stiffness model in SD rats and treated them with LIPUS (parameters: frequency 1.5 MHz, intensity 30 mW/cm^2^, and the duty cycle of 20%, 20 min per session, once a day, 5 days a week) for consecutive 2, 4, or 6 weeks. They found that the joint mobility in the treatment group was significantly better than that in the control group at weeks 4 and 6. Markers of synovial fibrosis, such as CTGF, TGF‐β, and Collagen I, were significantly lower in the LIPUS group than in the control group. The number of CD68^+^ cells, one of the inflammatory markers in the synovium, was high at 2 weeks and gradually decreased at 4 and 6 weeks, with a more pronounced decrease in the LIPUS treatment group. T. Zhou, Zhou et al. [61] immobilized the knee joint of rabbits for 6 weeks to establish the extended knee contracture model and followed applying LIPUS treatment (parameters: frequency 1.0 MHz, intensity 100 mW/cm^2^, 15 min per session, once per day) for 2 weeks. This research demonstrated that LIPUS significantly reduced the ROS level in the joint capsule and this effect partially inhibited the activation of TGF‐β1/Smad signaling, and further attenuated the joint capsule fibrosis to alleviate joint contracture.

In summary, the therapeutic effects of LIPUS on synovial inflammation center on reducing synovial fibrosis and suppressing inflammatory cytokine production by synovial macrophages (Table 2). However, some questions remain unresolved, such as: (1) its potential modulation of synovial macrophage polarization; (2) its impact on synovial lymphatic vessel function; and (3) its regulatory role in synovial sensory nerve activity for pain relief. These mechanisms still require rigorous investigation to advance clinical translation.

**TABLE 2:** The effects of LIPUS on synovial.

Author(Year)	Model	Parameter	Outcome	Ref.
Zhang et al. (2020)	Human THP‐1 monocytes and RAW 264.7 cells pretreated with LPS and ATP. Male C57 mice with DMM and a synovitis model using an air pouch.	1.5 MHz, 30 mW/cm^2^ for 20 min. 1.5 MHz, 30 mW/cm^2^, 20 min/d for 2w.	LIPUS inhibits the production of mature IL1B via SQSTM1dependent autophagic degradation of PKM2 in LPS‐ATP‐treated macrophages, which further ameliorate the synovial inflammation.	[55]
Feltham et al. (2021)	Rat IAF(Intra‐articular Fracture) OA model.	1.5 MHz, 30 mW/cm^2^, 20 min/d for 2w.	LIPUS attenuates leukocyte infiltration in the synovium, particularly CD3^+^ lymphocytes and CD68^+^ macrophages.	[60]
Liao et al. (2021)	OA patients FLSs. Male C57BL/6 mice DMM OA model.	1.5 MHz, 30 mW/cm^2^ for 20 min. 1.5 MHz, 30 mW/cm^2^, 20 min/d for 2w.	LIPUS alleviates synovial fibrosis in the knee joints, with the inhibition of synovial fibroblast proliferation and dependent on the Wnt/β‐catenin signaling pathway.	[56]
Itaya et al. (2018)	Right knee joints of male SD rats were immobilized with an internal, but extra‐articular, fixator at 150° of flexion to established a joint stiffness model.	1.5 MHz, 30 mW/cm^2^, 20 min/d for 2, 4, and 6w.	Markers of synovial fibrosis were lower in the LIPUS group than in the control group. The number of CD68^+^ cells in the synovium was high at 2 weeks and gradually decreased at 4 and 6 weeks after LIPUS.	[13]
Zhou et al. (2023)	Immobilized the left knee joint of rabbits for 6 weeks to establish an extended knee contracture model.	1 MHz, 100 mW/cm^2^, 15 min/d for 2w.	LIPUS reduces the level of ROS in the joint capsule and further inhibited the TGF‐β1/Smad signaling pathway, alleviating joint contracture.	[61]

### 4.3 LIPUS Improves Subchondral Bone Remodeling

**FIGURE 6:**
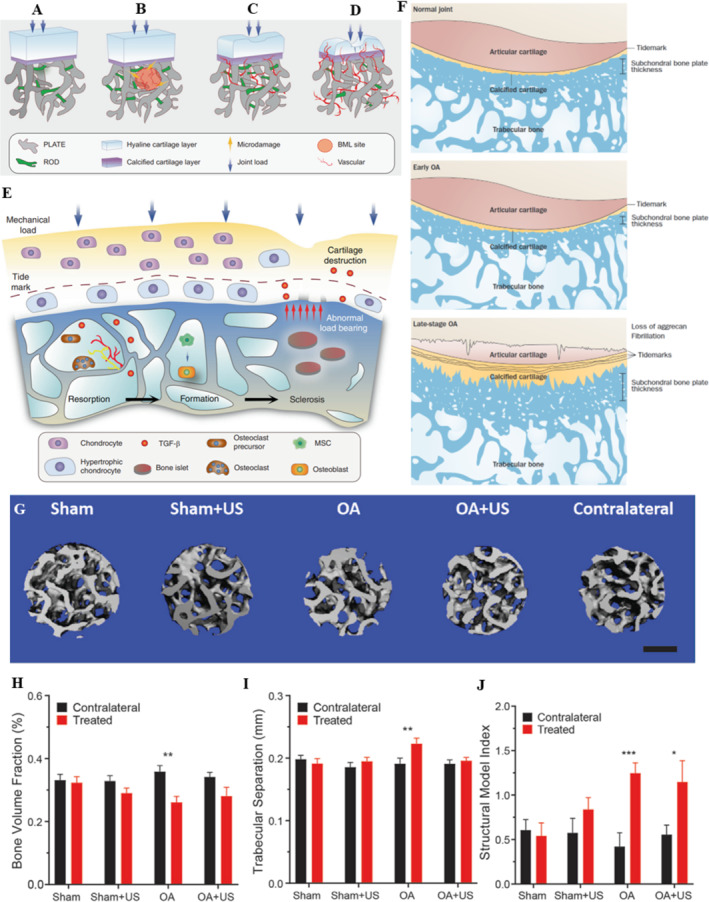
LIPUS improves subchondral bone remodeling. (A) Normal state of subchondral bone [62]; (B) Early stages of bone marrow injury (BML) and angiogenesis [62]; (C) Progressive stage combined with cartilage injury [62]; (D) Severe cartilage injury and vascular invasion in end stage [62]; (E) Schematic diagram of subchondral bone response to abnormal mechanical loads and accompanying cartilage destruction [62]; (F) Schematic diagram of progressive joint degeneration in OA [67]; (G) Segmented image of subchondral bone of each group in cylindrical regions. Scale bar: 500 μm [68]; Microarchitecture analyses of (H) bone volume fraction, (I) trabecular separation, and (J) structural model index [68]. Reproduced under terms of the CC‐BY license [62]. Copyright 2021, The Authors, published by Springer Nature. Reproduced with permission [67]. Copyright 2012, Springer Nature Limited. Reproduced with permission [68]. Copyright 2024, Elsevier (Singapore) Pte Ltd.

Likewise, the subchondral bone plays a significant role in OA, being adjacent to the articular cartilage and referring to the bone layer located beneath the cartilage. Anatomically, it can be subdivided into the upper subchondral bone plate (SBP) and the below subchondral trabecular bone. SBP is a porous calcified and compact plate crisscrossed by numerous blood vessels and nerve fibers. However, the subchondral trabecular bone is the cancellous bone structure located beneath the SBP, undergoing continuous bone remodeling [62]. It provides not only nutritional and mechanical support but also regulates the interaction with articular cartilage through signaling pathways, collectively maintaining joint homeostasis and directly or indirectly affecting cartilage metabolism [63, 64]. In early OA, the SBP gradually becomes more porous and thinner, accompanied by deterioration of the subchondral trabeculae and initial cartilage degeneration. In advanced OA, the calcified cartilage and SBP thicken [63, 65, 66]. This is accompanied by blood vessels and nerve branches extending into the cartilage from the subchondral bone, leading to cartilage destruction and joint pain. At the same time, subchondral bone manifests as cyst generation, bone marrow edema‐like lesions, and osteophyte formation (Figure 6) [69]. Among these, the TGFβ signaling is vital in maintaining the homeostasis of the subchondral bone [70, 71].

The effects of LIPUS on subchondral bone are shown in (Table 3). In detail, X. Li, Sun et al. [72] established a traumatic arthritis model using 5‐month‐old female SD rats with ACLT (Anterior Cruciate Ligament Transection) and treated the model with LIPUS (parameters: frequency 3 MHz, intensity 30 mW/cm^2^, duty cycle 20%, 20 min per session, once per day) for 6 weeks, with treatments administered 5 days a week. Micro‐CT scan results indicated that in the surgery‐only group, the thickness of the cartilage and the content of sulfated glycosaminoglycans decreased, while the thickness of the SBP increased. In contrast, the degree of subchondral bone sclerosis in the LIPUS treatment group was milder than that in the surgery alone. In a temporomandibular joint arthritis experiment with rabbits [73], LIPUS treatment (parameters: frequency 1 MHz, pulse repetition frequency 100 Hz, intensity 30 mW/cm^2^, duty cycle 20%, 20 min per session) for 3 or 6 weeks activated the TGF‐β1/Smad3 signaling pathway, inhibiting abnormal subchondral bone resorption and thereby improving the remodeling of the subchondral bone in the temporomandibular joint of rabbits. In a more recent study [68], a traumatic OA model was established using 4‐month‐old male SD rats with ACLT, and the model was treated with LIPUS (parameters: frequency 3.3 MHz, intensity 100 mW/cm^2^, duty cycle 20%, 20 min per session, once per day, 5 days a week) for 4 weeks. The results showed that LIPUS reduced the osteoclast activity in the subchondral bone of OA rats and alleviated the microstructural changes of osteoporosis in the subchondral bone of the surgery group. However, unlike cartilage and synovial studies, in vitro models for subchondral bone‐related studies have not yet been fully established. There is still a huge research gap on the specific mechanism by which LIPUS improves subchondral bone remodeling.

**TABLE 3:** Effects of LIPUS on subchondral bone.

Author(Year)	Model	Parameter	Outcome	Ref.
Li et al. (2019)	Female SD rats with ACLT (Anterior Cruciate Ligament Transection) to establish the OA model.	3 MHz, 30 mW/cm^2^, 20 min/d for 6w.	The degree of subchondral bone sclerosis in the LIPUS treatment group was milder than that in the surgery‐only group.	[72]
Yi et al. (2020)	The bilateral TMJ of rabbit was injected with 250μLof 10 mg/mL type II collagenase solution to establish the TMJOA model.	1 MHz, 30 mW/cm^2^, 20 min/d for 4w.	LIPUS improves the trabecular microstructure and histological characteristics of subchondral bone in rabbit TMJOA. It partially through TGF‐β1/Smad3 pathway.	[73]
Lee et al. (2024)	RAW 264.7 cells added RANKL to induce osteoclast differentiation. Male SD rats with ACLT (Anterior Cruciate Ligament Transection) to establish the OA model.	1 MHz, 30 mW/cm^2^, 20 min/d for 7d. 3.3 MHz, 0.1 W/cm^2^ peak intensity, and 20% duty cycle, 20 min/d for 4w.	LIPUS treatment has a suppressive effect on osteoclastogenesis, which may be linked to the suppression of sensory innervation in OA.	[68]

Currently, research on targeting subchondral bone with LIPUS for the treatment of OA is scarce and still in its infancy. Previous studies have primarily utilized LIPUS for the treatment of fractures, non‐unions [74], and even periodontal bone regeneration [75], all of which are indicative of its anabolic and angiogenic effects. However, abnormal ossification and angiogenesis of the subchondral bone are detrimental factors in the onset and progression of OA, which seems contradictory to the therapeutic mechanism of LIPUS. Is this related to the hypoxic environment of subchondral bone within the joint cavity, making it different from other peripheral bones? In OA progression, there is much remains unknown about the pathological changes in subchondral bone induced by LIPUS; further exploration is warranted.

### 4.4 LIPUS Alleviates Meniscus Injury

**FIGURE 7:**
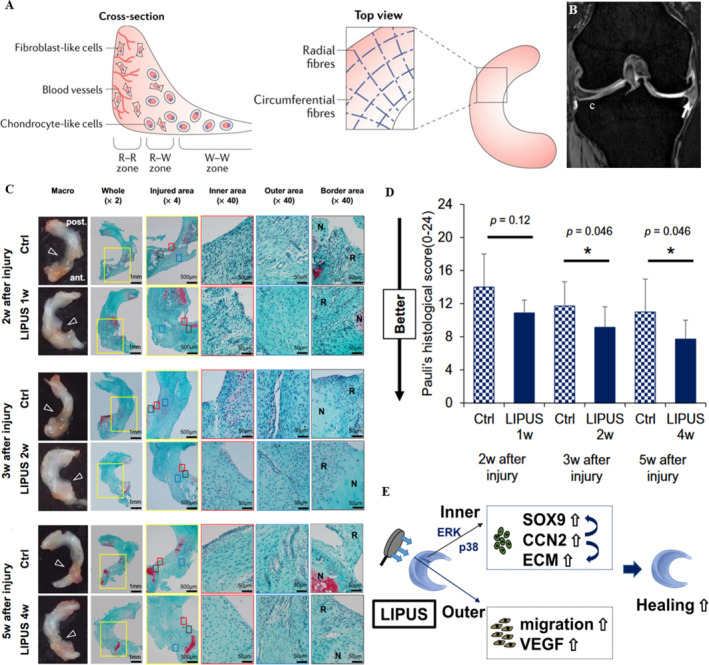
LIPUS relieves meniscal injuries. (A) Schematic diagram of meniscus structure [76]; (B) Coronary MRI of meniscus injury [77]; (C) Macroscopic and Safranin‐O staining analysis and (D) the Pauli's histological score of menisci at different time points after the injury for each group. White arrows: radial tear, N: native meniscus, R: repaired one [78]; (E) The diagrammatic of LIPUS healing meniscus [78]. Reproduced under terms of the CC‐BY license [76]. Copyright 2021, The Authors, published by Frontiers Media S.A. Reproduced under terms of the CC‐BY license [77]. Copyright 2022, The Authors, published by MDPI. Reproduced with permission [78]. Copyright 2019, Springer Nature.

As an essential intra‐articular structure, the meniscus is a semilunar fibrocartilage located between the femoral condyles and tibial plateau. Its biomechanical functions include load‐bearing, shock absorption, and joint lubrication during knee movement [76]. Anatomically, the meniscus comprises three distinct regions: the vascularized red‐red (R‐R) zone, the transitional red‐white (R‐W) zone, and the avascular white‐white (W‐W) zone (Figure 7A) [76]. Notably, the W‐W zone shares histological similarities with articular cartilage and exhibits limited regenerative capacity due to its avascular nature. Meniscal tears contribute to and accelerate OA progression by disrupting load distribution, impairing pressure cushioning, and compromising knee stability (Figure 7B) [77, 79]. A large‐scale data analysis further identified meniscal morphology as a critical risk factor for knee OA development [80].

Current research on the use of LIPUS for treating meniscal injuries is limited (Table 4). Kamatsuki et al. [78] performed an anterior horn resection of the lateral meniscus of rats and treated them with LIPUS for 1, 2, or 4 weeks after surgery (parameters: frequency 1.5 MHz, intensity 60 mW/cm^2^, 20 min per session, once per day), combined with in vitro experiments using meniscal cells from OA patients obtained during joint replacement surgery (parameters: frequency 3.0 MHz, intensity 60 mW/cm^2^, duration 20 min). This study deduced treatment mechanisms corresponding to the structural zones of the meniscus. On the one hand, in the avascular W‐W zone, LIPUS promotes the expression of CCN2 and SOX9 in medial meniscal cells through the MAPK signaling pathway, thereby promoting the formation of their cartilaginous matrix. In the vascular R‐R and R‐W zones, LIPUS promotes the expression of CCN2 in lateral meniscal cells and further enhances cell proliferation and angiogenesis, playing a role in the repair of meniscal injuries through both aspects (Figure 7C–E). Given the unique structure of the meniscus, the biphasic nature of LIPUS treatment for meniscal injuries may provide a new perspective for the treatment of arthritis. Another study also showed that LIPUS promotes the proliferation and migration of chondrocytes in 3D culture and upregulates the expression of proteoglycans and collagen II, recommending that the combination of LIPUS and 3D hybrid scaffolds may become a new strategy to promote the regeneration of meniscus [81].

**TABLE 4:** Effects of LIPUS on meniscus.

Author(Year)	Model	Parameter	Outcome	Ref.
Kamatsuki et al. (2019)	Meniscal cells from OA patients obtained during joint replacement surgery. Rats underwent anterior horn resection of the lateral meniscus to establish the OA model.	3 MHz, 60 mW/cm^2^, for 20 min. 1.5 MHz, 60 mW/cm^2^, 20 min/d for 1, 2 or 4w.	LIPUS promotes the expression of CCN2 and SOX9 in medial meniscal cells through the MAPK pathway while promotes the expression of CCN2 in lateral meniscal cells and further enhances cell proliferation and angiogenesis.	[78]
Babaei et al. (2022)	The chondrocytes were seeded on the 3D hybrid scaffolds.	1 MHz, 100, 200, and 300 mW/cm^2^, for 20 min.	The combination of LIPUS treatment and 3D hybrid scaffolds can be considered as a valuable treatment for meniscus regeneration.	[81]

### 4.5 LIPUS Reduces the Sensory Nerve Innervation

**FIGURE 8:**
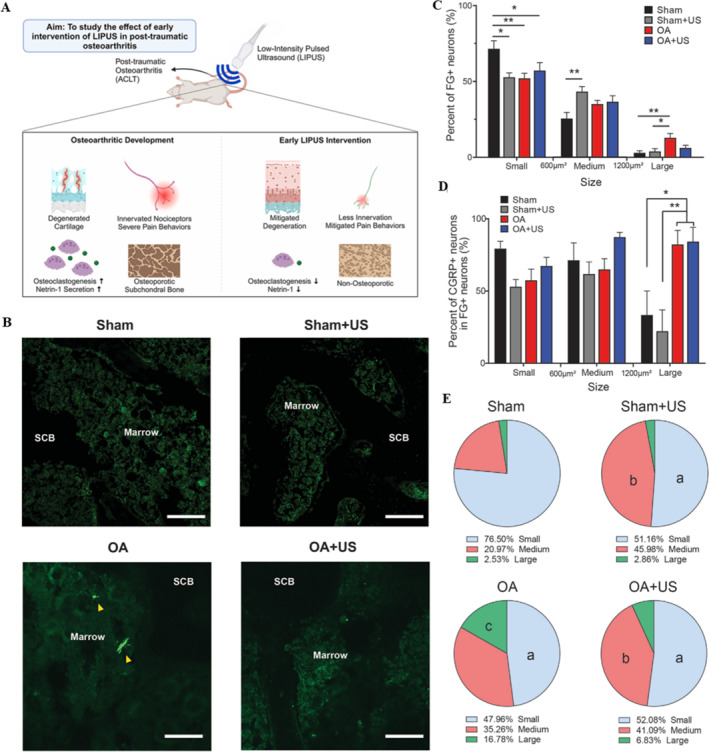
LIPUS reduces sensory nerve innervation. (A) The diagrammatic drawing of LIPUS improving subchondral bone remodeling and reducing sensory innervation. (B) Immunofluorescence of CGRP‐positive (arrowhead) in the subchondral bone marrow. Scale bar: 100 μm. (C) The statistic of FG‐positive neurons in each subgroup, small (< 600 μm^2^), medium (> 600 μm^2^, < 1200 μm^2^), and large (> 1200 μm^2^); (D) the statistic of CGRP‐positive neurons in each subgroup of neurons; (E) the proportion of each subgroup of neurons in different experimental groups [68]. Reproduced with permission [68]. Copyright 2024, Elsevier (Singapore) Pte Ltd.

OA is a chronic degenerative joint disorder associated with debilitating pain and is also the main reason for patients seeking medical attention. To further elucidate the pain mechanism of OA, emerging preclinical evidence has delineated distinct patterns of sensory neuroplasticity in OA pathophysiology. Neuroanatomical tracing studies utilizing calcitonin gene‐related peptide (CGRP), a canonical marker of peptidergic nociceptors, revealed significant reorganization of articular innervation across murine OA models. Quantitative immunohistochemical analyses demonstrated that 70% of dorsal root ganglion (DRG) neurons exhibit CGRP immunoreactivity, with soma topographically organized according to joint innervation territories [82]. Notably, chemical denervation models (monoiodoacetate [MIA]‐induced OA) and surgical instability models (destabilized medial meniscus [DMM]) exhibit increases in CGRP nerve fiber density compared with sham controls [83, 84]. A recent study established a traumatic OA model in 4‐month‐old male SD rats using ACLT. The rats were treated with LIPUS (parameters: frequency 3.3 MHz, intensity 100 mW/cm^2^, duty cycle 20%, 20 min per session, once per day, 5 days per week) for a duration of 4 weeks post‐surgery. The results demonstrated that LIPUS significantly alleviated pain and joint degeneration in the rats, which may be associated with its capacity to ameliorate the microstructure of subchondral bone and diminish sensory nerve innervation (Figure 8) [68]. These data demonstrated that LIPUS significantly attenuates osteoclastogenesis by concomitantly suppressing netrin‐1 secretion and nociceptive nerve fiber density in subchondral bone, and established a novel biophysical paradigm for LIPUS‐mediated analgesia in OA.

Collectively, preclinical studies demonstrate that LIPUS attenuates arthritis progression via five core mechanisms: (1) enhancing cartilage matrix anabolism while suppressing catabolism; (2) modulating synovial inflammation to reduce pain and stiffness; (3) optimizing subchondral bone remodeling; (4) preventing meniscal degeneration; and (5) regulating nociceptive signaling in sensory nerves. These therapeutic effects are mediated through molecular pathways including NF‐κB, FAK, p38‐MAPK, ERK1/2, and PI3K/Akt, which coordinate cellular processes such as autophagic flux, motility, and proliferation.

## 5 The Mechanism of LIPUS Treating OA Indirectly

**FIGURE 9:**
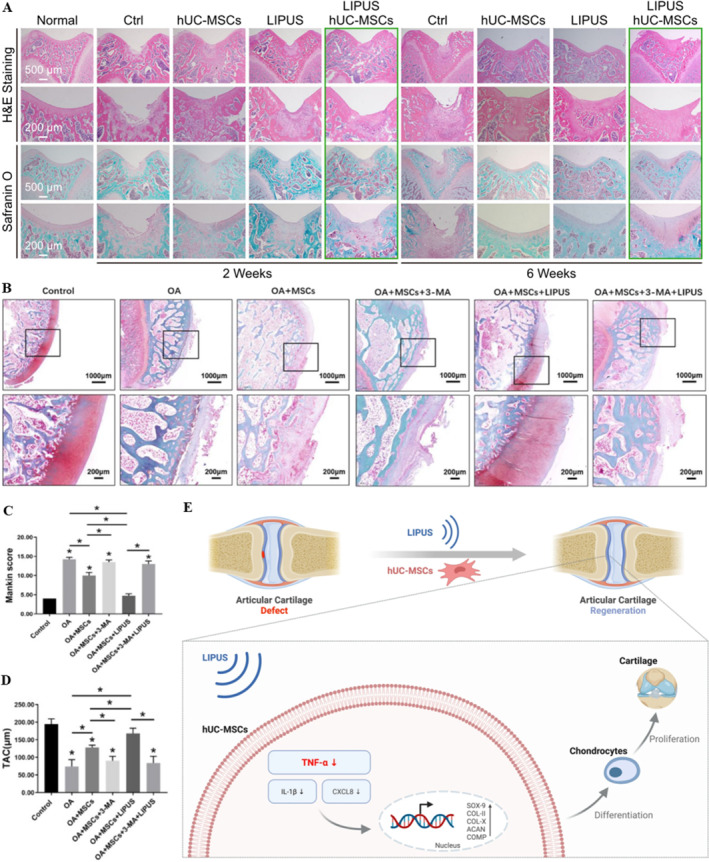
LIPUS alleviates arthritis indirectly. (A) Respective H&E staining and Safranin O‐fast green staining of the paraffin sections of the knee joint defect showing the healing effect of LIPUS stimulation, scale bar: 500, 200 um [85]; (B) Safranin‐O/fast green staining showing the cartilage morphological changes under a microscope, scale bars ¼ 1000 mm, 200 mm [86]; (C) The statistic of Mankin scores of each group [86]; (D) The statistic of TAC thickness (mm) [86]; (E) Schematic of LIPUS combined with hUC‐MSC for cartilage regeneration [85]. Reproduced under terms of the CC‐BY license [85]. Copyright 2023, The Authors, published by BioMed Central. Reproduced with permission [86]. Copyright 2021, The Authors, published by SAGE Publications Inc.

In addition to the direct use of LIPUS in the treatment of OA, there are also other cells or factors that can be used as a medium to make LIPUS exert indirect therapeutic effects. For example, LIPUS was utilized to stimulate HUC‐MSCs and C28/I2 cells in vitro. A rat articular cartilage injury model was established to further conduct HUC‐MSC transplantation and to use LIPUS stimulation in vivo. The experimental data demonstrated that LIPUS stimulation with optimized parameters significantly upregulated the expression of mature cartilage‐specific genes (e.g., COL2A1, ACAN) and extracellular matrix proteins, while concurrently suppressing TNF‐α gene expression in hUC‐MSCs. This regimen also exhibited pronounced anti‐inflammatory effects in C28/I2 chondrocyte cultures. Furthermore, combinatorial therapy involving hUC‐MSC transplantation and LIPUS application achieved substantial articular cartilage regeneration in rat defect models, as evidenced by histological and biomechanical assessments. In summary, LIPUS stimulation achieves articular cartilage regeneration based on HUC‐MSC transplantation by inhibiting the TNF signaling pathway (Figure 9A, E) [85]. Wang, Lin et al. [87] found that LIPUS could up‐regulate the chondrogenic differentiation of MSCs, as evidenced by the upregulation of ECM and chondrogenic genes such as SOX9. The maximum effect was observed at an intensity of 50 mW/cm^2^. These effects were augmented and inhibited by autophagy inhibitors and agonists, respectively, demonstrating that LIPUS promotes chondrogenesis through autophagy of MSCs. Additionally, Xia, Wang, Wang et al. [86] injected a suspension of MSCs into the joints of OA rats modeled by ACLT and subjected the rat joints to LIPUS treatment (parameters: frequency 3 MHz, intensity 20, 30, 40, 50 mW/cm^2^, duty cycle 20%, 20 min per session, once per day, 4 sessions/10 days) and found it to be effective in articular cartilage repair. Related in vitro experiments for mechanistic studies indicated that LIPUS could upregulate the migration mediated by autophagy in mesenchymal stem cells, thereby playing a role in cartilage repair, with the effect being most pronounced at an intensity of 50 mW/cm^2^ (Figure 9B–D). Notably, LIPUS stimulation significantly enhances extracellular vesicle (EV) secretion by bone marrow‐derived mesenchymal stem cells (BMSCs). Comparative analyses reveal that EVs from LIPUS‐treated BMSCs exhibit markedly elevated anti‐inflammatory activity compared to those secreted by untreated controls. These findings collectively suggest that LIPUS represents a viable non‐invasive approach to enhance both the yield and anti‐inflammatory efficacy of BMSC‐derived EVs [88].

These studies collectively leverage MSCs' stem cell properties as a mechanistic bridge to augment LIPUS‐induced chondrogenic differentiation potential and amplify its therapeutic efficacy in arthritis. Notably, the ability of LIPUS to enhance exosome biogenesis has emerged as an active research focus. Two critical questions warrant further investigation: (1) extending this paradigm to non‐MSC populations for broader regenerative applications, and (2) determining whether LIPUS facilitates cellular exosome uptake—a process pivotal for intercellular communication.

## 6 The Combination of LIPUS and Biomaterials for OA Treatment

**FIGURE 10:**
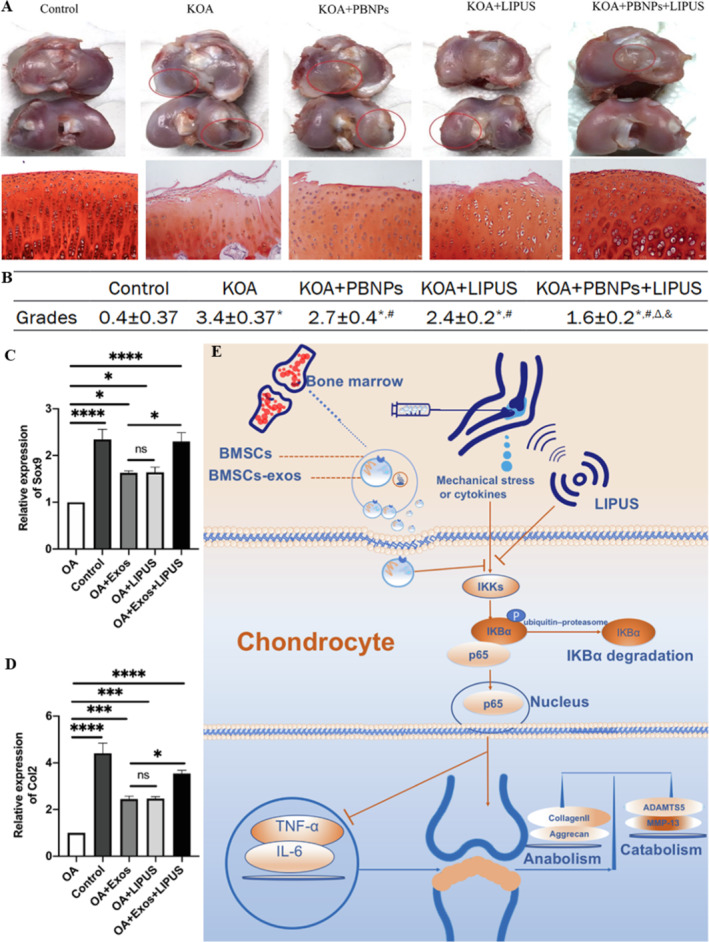
LIPUS combined with biomaterials for the treatment of OA. (A) Macroscopic observation of the cartilage tissue in each group, circles indicate lesion areas (upper). Safranin O staining of the species in each group (under) [91]; (B) Cartilage lesion grades measured by macroscopic examination (*n* = 5) [91]; Protein expression of Sox9 (C) and Col2 (D) were measured by western blot [90]; (E) A mechanism diagram of LIPUS‐mediated BMSC‐derived exosomes maintaining cartilage homeostasis [90]. Reproduced with permission [90]. Copyright 2021, The Authors, published by Elsevier. Reproduced with permission [91]. Copyright 2021, e‐Century Publishing Corporation.

With the advancement of interdisciplinary approaches, research on the application of LIPUS in the treatment of OA has expanded rapidly. Researchers are increasingly exploring the combination of LIPUS with other therapeutic modalities for OA management. Detailly, C. H. Chen, Kuo et al. [89] conducted a study using a spontaneous OA model in guinea pigs aged 6 months and found that 8 weeks of LIPUS treatment (parameters: frequency 3 MHz, intensity 100 mW/cm^2^, duty cycle 20%, 20 min per session, once per day, 3 days per week) combined with liposome‐encapsulated rapamycin (intra‐articular injection, twice a week) was more effective than monotherapy with either rapamycin intra‐articular injection, liposome‐encapsulated rapamycin, or LIPUS alone. However, further in‐depth investigation into the underlying mechanisms was not pursued. Furthermore, Q. Liao, Li et al. [90] utilized a rat ACLT model of traumatic arthritis and initiated treatment with LIPUS (parameters: frequency 1.5 MHz, intensity 30 mW/cm^2^, duty cycle 20%, 20 min per session, once per day, 7 days per week) combined with intra‐articular injection with exosomes derived from BMSs (twice a week) beginning at 6 weeks post‐surgery. The therapeutic evaluation showed that LIPUS further enhanced the effects of cartilage matrix synthesis and inflammation inhibition by exosome injection. In vitro experiments indicated that this effect was partially mediated through the NF‐κB signaling pathway (Figure 10C–E). Moreover, Xia et al. further discovered that LIPUS enhances the therapeutic efficacy of BMSCs in OA cartilage repair by modulating autophagy‐mediated exosome release [92]. A study by Zuo et al. [91] combined Prussian blue nanoparticles with LIPUS, and rabbits were subjected to ACLT followed by LIPUS irradiation (acoustic intensities of 60 mW/cm^2^, duty ratio of 20%, central frequency of 1.5 MHz, and repetition frequency of 1 KHz) for 20 min per day, 5 days per week for 6 weeks (Figure 10A,B). They found that this combination reduced chondral damage in New Zealand rabbits with knee OA through the activation of the PI3K/Akt/mTOR pathway, with effects superior to those of LIPUS alone, primarily related to the effective scavenging of ROS by Prussian blue nanoparticles in inflammatory environments. In a recent study, Song et al. [93] conducted a comparative evaluation of LIPUS, nano‐hydroxyapatite (NHA), and their combined therapeutic efficacy in rabbit models of OA. The experimental data revealed that the NHA‐LIPUS combined intervention demonstrated synergistic therapeutic efficacy, achieving superior cartilage regeneration outcomes compared to either NHA or LIPUS monotherapy. This phenomenon may be associated with the downregulation of pERK1/2 and p38 expression levels in chondrocytes. Additionally, four joint friction parameters (friction coefficient, oscillation number, maximum peak oscillation value, and exponential curve slope) were employed to evaluate the combined therapeutic effects of LIPUS and platelet‐rich plasma (PRP). The results demonstrated that PRP exhibited no significant superiority over LIPUS in reducing joint friction or enhancing the biomechanical properties of guinea pig knee joints. Notably, LIPUS provides a non‐invasive therapeutic approach compared to the invasive nature of PRP injection. Furthermore, the combined application of LIPUS and PRP showed no additional benefits over LIPUS monotherapy in improving knee joint cartilage biomechanical properties or mitigating joint friction [94]. Specifically, the therapeutic mechanism of LIPUS involves pressure wave propagation that directly modulates chondrocyte activity via integrins and membrane‐bound receptors [12]. Ultrasonic pressure waves activate specific signaling pathways associated with improved cartilage surface regularity [95]. These combined effects suggest that LIPUS may enhance cartilage's mechanical resistance to compressive forces. Additionally, its potential to upregulate lubricin expression and optimize articular lubrication factors could contribute to reduced joint friction, which needs further investigation.

Current LIPUS‐based therapeutic strategies often integrate intra‐articular injections in preclinical models. However, repeated injections risk iatrogenic damage to periarticular tissues (e.g., dermis and synovium) and may paradoxically exacerbate synovitis. The clinical viability of this combinatorial approach—particularly its capacity to elicit synergistic therapeutic effects exceeding individual modalities—requires systematic validation. Notably, ultrasound‐targeted therapy has demonstrated enhanced precision in disease modulation through spatiotemporal energy focusing. Furthermore, ultrasound‐responsive piezoelectric platforms enable mechanochemical transduction, potentially revolutionizing controlled drug delivery and on‐demand release of bioactive agents [96]. For instance, ultrasound has shown potential for non‐invasively breaching the blood‐brain barrier, enhancing targeted drug delivery by safely and selectively permeating the barrier to treat neurological disorders [97] and brain tumors [98]. Given the relatively weak focusing effects of LIPUS, experimental studies exploring its combination with biomaterials for drug delivery remain scarce, whereas current research has predominantly focused on low‐intensity focused ultrasound (LIFU) coupled with biomaterials. For example, W. Han et al. [99] synthesized an FA@IL‐10@HMs ultrasound contrast microsphere system, a novel biomaterial capable of sustainably targeting inflammatory macrophages while enabling contrast‐enhanced imaging and ultrasound‐triggered release of anti‐inflammatory agents, thereby establishing a novel theranostic platform for monitoring and managing synovitis in osteoarthritis.

Emerging therapeutic strategies increasingly integrate advanced biomaterials and novel technologies to address arthritic pathologies, offering valuable insights for targeted treatment design. Notably, Yang, Sun et al. [100] developed bioinspired methacrylated hyaluronic acid (HAMA) microcarriers functionalized with 2‐methacryloyloxyethyl phosphorylcholine (MPC) for diclofenac sodium (DS) delivery, demonstrating potent anti‐inflammatory effects and enhanced therapeutic outcomes in both cellular and animal models of OA. This biomimetic strategy was derived from the superlubricated ice surface mechanism —specifically, the interfacial bound water layer that reduces friction—enabling the fabrication of MPC‐modified HAMA particles via microfluidic electrospray. The optimized system exhibited synergistic biomechanical properties, including exceptional lubricity and optimal mechanical stability, thereby advancing intra‐articular drug delivery systems for osteoarthritis management. They also fabricated magnetic polysaccharide hydrogel‐based microcarriers for synergistic OA therapy. These microcarriers comprised HAMA and chondroitin sulfate (CSMA)—fabricated via an integrated microfluidic electrospray and cryogelation strategy. Magnetic nanoparticles, engineered with spiny architectures for efficient stem cell‐derived exosome (Exo) capture, were co‐encapsulated with the anti‐inflammatory agent DS within the hydrogel matrix. Sustained co‐release of DS and Exo from the microcarriers exhibited a synergistic therapeutic effect, significantly alleviating OA‐associated inflammation while promoting cartilage matrix regeneration [101]. Given the distinct roles of platelet‐derived growth factor BB (PDGF‐BB) in stem cell recruitment and transforming growth factor β3 (TGF‐β3) in chondrogenic differentiation, these cytokines were co‐encapsulated within inverse opal microcarriers as a dual‐factor delivery platform. Sun et al. [102] demonstrated that PDGF‐BB/TGF‐β3‐loaded inverse opal microcarriers significantly amplify therapeutic efficacy, highlighting the potential of this multifunctional cytokine delivery system for targeted osteoarthritis intervention.

Therefore, developing alternative non‐invasive therapeutic modalities to synergistically augment LIPUS efficacy —through novel biomaterial integration to amplify mechanobiological effects or refine drug targeting precision—represents a critical frontier in OA management. The aforementioned advancements in bioengineered materials and emerging technologies offer transformative conceptual frameworks, such as integrating LIPUS with advanced biomaterial systems to either enhance spatiotemporal targeting specificity or achieve spatiotemporal control over therapeutic agent release and biodegradation kinetics.

## 7 Clinical Study of LIPUS in Treating OA

Although preclinical evidence robustly establishes LIPUS's therapeutic potential for attenuating OA progression in animal and cellular models, clinical translation remains limited by a paucity of human trials and inconsistent efficacy outcomes.

In detail, Yegin et al. [103] conducted a 1‐month LIPUS treatment with follow‐up for patients with OA, revealing that LIPUS could effectively reduce pain and improve knee joint function during the first 2 weeks of treatment, but there was no statistically significant difference in pain and function improvement at the 1‐month time point. In a randomized, double‐blind clinical controlled trial [35], 96 patients with OA were randomly divided into the LIPUS treatment group and the control, with LIPUS stimulation (parameters: frequency 1 MHz, intensity 1 W/cm^2^, duty cycle 20%, 10 min per session, once per day, three days per week) conducted for the first eight weeks, followed by a 4‐week follow‐up. Ultimately, data from 75 patients were included in the statistics, showing that LIPUS treatment could significantly reduce pain and enhance daily living abilities in patients. Another randomized, double‐blind, placebo‐controlled trial [30], found that 10 days of FLIPUS (Focused Low‐intensity Pulsed Ultrasound) (parameters: frequency 0.6 MHz, intensity 100 mW/cm^2^, duty cycle 20%, 20 min per session, once per day) could provide better outcomes than the control group in terms of ROM scores, VAS scores, and walking speed for patients with OA. There are also meta‐analysis [29, 104] indicating that LIPUS, as a non‐invasive treatment for OA, can alleviate pain and to some extent improve joint function and ROM scores. A clinical study in the oral field found that LIPUS could effectively alleviate pain in patients with myofascial pain and temporomandibular joint synovitis; however, myofascial pain is more sensitive to LIPUS [105]. In recent years, there have been some controversial studies on its efficacy. Jo et al. [106] found in a clinical trial that after 4 weeks of LIPUS treatment (parameters: frequency 1.0 MHz, intensity 600 mW/cm^2^, 30 min per session, once per day, 5 days per week), patients with knee arthritis experienced increased joint function, and quality of life, but contrary to numerous basic studies, the results of joint MRI examinations suggested that the thickness of their articular cartilage did not significantly increase. Moreover, Kim et al. [107] assessed the safety and efficacy of LIPUS combined with TENS in knee osteoarthritis in a clinical study and found that the combination of LIPUS and TENS did not provide better pain relief and functional improvement than TENS therapy alone, and for cartilage regeneration, it was speculated that the LIPUS combination group would have better outcomes, but it did not show the expected advantage. Compared with LIPUS combined with exercise, High‐intensity Laser Therapy combined with exercise achieved better results in terms of pain, knee joint mobility, proprioceptive accuracy, and functional impairment, and both combined treatments were superior to exercise therapy alone [36]. In the latest randomized, double‐blind, controlled trial, the study recruited 200 KOA patients. They were randomly assigned in a 1:1 ratio to either a LIPUS treatment group or a control group. The consecutive 2‐week LIPUS treatment consisted of five sessions per week. At 1 month post‐intervention, the Western Ontario and McMaster Universities Osteoarthritis Index (WOMAC) scores were assessed as the primary outcome. Secondary outcomes included the Numerical Rating Scale (NRS) for pain intensity, Lequesne Index for functional disability, and knee joint range of motion. The results demonstrated significant efficacy of LIPUS in alleviating pain and enhancing functional capacity in patients with KOA [108].

In conclusion, the clinical efficacy of LIPUS for OA remains controversial. The discrepancies may be attributed to the following factors: (1) The assessment criteria in clinical studies have predominantly utilized pain‐ or function‐related scales, which are heavily dependent on subjective patient perceptions and lack objective quantitative measures; (2) Substantial heterogeneity exists among clinical populations, where variables such as disease severity, age, gender, and body weight may introduce variability in outcomes; (3) Considerable variations exist in treatment protocols across studies, including differences in treatment duration, frequency, and operator techniques; (4) Evidence suggests that temperature, thickness, or density of the coupling medium may affect acoustic energy transmission [109], consequently influencing therapeutic outcomes through environmental factors and patients' skin and musculoskeletal conditions. Therefore, determining the optimal parameters and treatment duration for LIPUS therapy in OA remains a critical challenge, with the establishment of standardized treatment protocols representing the highest priority.

Notably, no adverse reactions associated with LIPUS therapy have been reported in clinical studies to date. Given the high global prevalence of OA, LIPUS emerges as a promising cost‐effective, non‐pharmacological, and non‐invasive therapeutic strategy for OA management.

## 8 Summary and Prospects

### 8.1 The Dose‐Response Relationship of LIPUS Parameters Still Needs Further Optimization and Control

Comparative analyses of LIPUS applications in arthritis research reveal significant heterogeneity in treatment parameters (e.g., intensity, frequency, duration) across preclinical and clinical studies. This variability stems from divergent experimental designs, including species‐specific OA models, cell culture systems, and device specifications. Mechanistically, LIPUS‐induced mechanobiological responses depend critically on pulse repetition frequency, acoustic intensity, duty cycle, and exposure duration. Key experimental confounders—such as sensor‐target positioning, cell‐transducer distance, coupling medium properties (thickness/viscosity), and culture conditions—further modulate these effects [109]. Standardizing experimental protocols is therefore essential to ensure reproducibility. Optimizing LIPUS parameters for clinical translation remains an urgent priority.

### 8.2 LIPUS Combined With Biomaterials has Broad Application Prospects

The research on LIPUS for the treatment of osteoarthritis (OA) primarily focuses on its biological effects such as promoting the synthesis of cartilage matrix and anti‐inflammatory actions. As a physical therapeutic agent, LIPUS mainly exerts its effects through mechanical means, yet the specific mechanisms of how it achieves mechanical transduction with biological systems remain unclear. With the promotion of LIPUS in the treatment of osteoarthritis, more and more studies have shown that the combination of LIPUS with nonsteroidal anti‐inflammatory drugs for oral or external use, as well as joint cavity drug injection, exercise training, and other methods, is more effective than using LIPUS alone for treatment. Exploring new materials to combine with LIPUS to enhance its mechanical transduction effect effects is also an emerging avenue of research. Due to the growing demand for implantable devices that interact directly with biological tissues, there has been a significant shift toward in vivo applications. The development of biodegradable piezoelectric materials has greatly reduced the risks and inconveniences associated with traditional piezoelectric implants, offering more possibilities for the combination of ultrasound and biomaterials. Although biodegradable piezoelectric biomaterials have significant advantages, they face challenges in maintaining sufficient piezoelectric response and stability to support the entire expected lifespan of an ultrasound power transfer (UPT) system. Moreover, the degradation rate must be carefully balanced through material design and encapsulation strategies to ensure the device operates normally until it is no longer needed. Degradation that is too rapid or too slow can lead to premature device failure or extended tissue exposure, potentially triggering adverse biological responses. Therefore, enhancing power transfer efficiency, precise degradation triggering, optimizing electrode materials, and determining appropriate use scenarios are crucial for the continued acceptance of UPT systems [110]. Ultrasound‐driven biomedical innovations are attracting significant research attention. Notably, acoustically actuated microrobots enable non‐invasive surgical precision and spatiotemporally controlled drug delivery in vivo. This paradigm synergizes with advanced biofabrication techniques to engineer hierarchical tissue architectures and implant functional biomaterials with micron‐level accuracy. Such advancements position ultrasound at the forefront of regenerative therapies, smart diagnostics, and programmable minimally invasive interventions.

### 8.3 The Application of LIPUS in Clinical Practice Still Needs to Be Promoted and Explored

While LIPUS demonstrates robust therapeutic outcomes in preclinical OA models, clinical applications remain largely confined to pain relief and ROM improvement, with mechanistic insights remaining poorly characterized. Three critical gaps persist: (1) decoding the mechanotransduction pathways linking LIPUS‐derived biophysical stimuli to intracellular responses; (2) elucidating disease‐specific therapeutic mechanisms beyond OA; and (3) establishing evidence‐based protocols for dosimetry optimization (intensity/frequency/duration) and treatment regimen standardization. As a cost‐effective non‐invasive modality, LIPUS holds transformative potential—systematic mechanistic and translational research could redefine its role in precision rehabilitation, ultimately offering a paradigm shift in OA management.

## Author Contributions

M.T. Guan, X.Y. Zhang, and X.H. Li contributed equally to this work and are the first co‐authors. All authors were involved in drafting the article or revising it critically for important intellectual content, and all authors approved the final version to be published. M.T. Guan and X.Y. Zhang researched data for the article, discussed the content, and wrote the manuscript before submission. X.H. Li translated the manuscript and managed tables. X.Y. Han and D.Q. Bai reviewed and edited the manuscript. B. Liao, W. Han, J.D. Tan, and Z.J. Wang joined the discussion of content and designed the figures. L.C. Wang helped in improving the language. J.L. Shen, X.Y. Han, and D.Q. Bai applied the funding acquisition and project administration.

## Conflicts of Interest

The authors declare no conflicts of interest.
